# Inhibition of Tankyrases Induces Axin Stabilization and Blocks Wnt Signalling in Breast Cancer Cells

**DOI:** 10.1371/journal.pone.0048670

**Published:** 2012-11-07

**Authors:** Renyue Bao, Tania Christova, Siyuan Song, Stephane Angers, Xiaojun Yan, Liliana Attisano

**Affiliations:** 1 Department of Biochemistry, Donnelly Centre for Cellular and Biomolecular Research, University of Toronto, Toronto, Ontario, Canada; 2 College of Animal Sciences, Zhejiang University, Zhejiang, Hangzhou, China; 3 Department of Pharmaceutical Sciences, Leslie Dan Faculty of Pharmacy, University of Toronto, Toronto, Ontario, Canada; Northwestern University Feinberg School of Medicine, United States of America

## Abstract

Constitutive Wnt signalling is characterized by excessive levels of β-catenin protein and is a frequent occurrence in cancer. APC and Axin are key components of the β-catenin destruction complex that acts to promote β-catenin degradation. The levels of Axin are in turn controlled by tankyrases, members of the PARP-family of poly-ADP-ribosylation enzymes. In colorectal cancer cells, which typically harbor APC mutations, inhibition of tankyrase activity promotes Axin stabilization and attenuates Wnt signalling. Here, we examined the effect of inhibiting tankyrases in breast cancer cells with normal APC. We show that application of the small molecule tankyrase inhibitor, XAV939 or siRNA-mediated abrogation of tankyrase expression increases Axin1 and Axin2 protein levels and attenuates Wnt-induced transcriptional responses in several breast cancer lines. In MDA-MB-231 cells, inhibiton of tankyrase activity also attenuate Wnt3a induced cell migration. Moreover, in both MDA-MB-231 and colorectal cancer cells, XAV939 inhibits cell growth under conditions of serum-deprivation. However, the presence of serum prevents this growth inhibitory effect, although inhibition of Wnt-induced transcriptional and migratory responses was maintained. These results indicate that stabilization of Axin by inhibition of tankyrases alone, may not be an effective means to block tumor cell growth and that combinatorial therapeutic approaches should be considered.

## Introduction

Wnt signalling plays a fundamental role during development and in adult homeostasis and is inappropriately activated in many types of cancers [1], [2], [3]. Canonical Wnt signals are mediated by β-catenin, a key downstream effector of the pathway, whose degradation is controlled by a complex consisting of the tumor suppressor Adenomatous polyposis coli (APC), Axis Inhibitor (Axin), and Glycogen Synthase Kinase 3 (GSK3). In the absence of Wnt, cytosolic β-catenin levels are kept low by the destruction complex. Wnt ligand activates the pathway by inducing stabilization of β-catenin and thereby promoting β-catenin nuclear accumulation. In the nucleus, β-catenin interacts with transcription factors of the LEF/TCF (Lymphoid enhancer-binding factor 1/T-cell factor) class and induces expression of TCF responsive target genes, such as c-Myc, cyclin D, Axin2 and Nkd1 [4], [5], [6], [7].

In human cancers, mutations and truncations in APC are linked to the familial adenomatous polyposis (FAP) coli syndrome and are found in the majority of sporadic colon carcinomas [8]. These alterations in APC or alternatively, mutations in β-catenin result in deregulation of β-catenin turnover and increase β-catenin/TCF signalling in colon cancer [1], [2], [3], [9]. In breast cancer, mutations in APC or β-catenin are rare, but elevated levels of β-catenin are prevalent and this aberrant activity is thought to promote mammary carcinogenesis [10], [11]. Indeed, increased β-catenin activity is correlated with poor prognosis in breast cancer patients [12] and in animal studies, activation of Wnt/β-catenin signalling by overexpression of Wnts or a stabilized form of β-catenin, leads to mammary tumourigenesis [10], [11]. Moreover, Wnt signalling plays an important role in stem cell-self renewal and thus may promote the growth of cancer stem cells, which are thought to drive tumorigenesis in a variety of solid tumors [10], [13].

The efficient assembly of the multi-protein destruction complex is dependent on the steady-state levels of its principal constituents. Axin has been reported to be the concentration-limiting factor in regulating the efficiency of the β-catenin destruction complex [14], [15]. Overexpression of Axin induces β-catenin degradation in cell lines expressing truncated APC [16], [17], [18], therefore, it appears that Axin protein levels are strictly controlled to ensure proper Wnt pathway signalling. There are several processes that control Axin levels and in fact, Wnt signalling itself regulates the level of Axin at several steps, with Axin2 being a major transcriptional target of the β-catenin–TCF complex and Wnt signalling promoting the degradation of Axin [19], [20].

Two separate studies involving chemical screens for Wnt pathway inhibitors identified compounds XAV939 and IWR-1 that promote Axin stabilization and thereby attenuate Wnt signalling [21], [22]. Additional analysis focussed on XAV939 demonstrated that both compounds act as inhibitors of TRF-1-interacting ankryin-related ADP-ribose polymerases (tankyrases/TNKS) of which there are two, tankyrases 1 and 2 [21]. More recent studies have identified other tankyrase inhibitors with divergent chemotypes, some of which have been characterized and similarly shown to promote stabilization of Axin *in vitro* and *in vivo* [1], [23], [24], [25], [26], [27], [28]. Mechanistically, tankyrases were shown to interact with a highly conserved domain of Axin and promote ubiquitination and degradation of Axin in a PARsylation dependent manner [21], [23]. Further insights were provided by means of RNAi screening, in which researchers identified the RNF146 RING-type ubiquitin E3 ligase as a positive regulator of Wnt signalling that cooperates with tankyrases to maintain low steady-state levels of Axin proteins [29], [30]. RNF146 was shown to directly interact with poly(ADP-ribose) through its WWE domain, and to mediate degradation of PARsylated proteins. Altogether, these studies describe a novel mechanism to promote β-catenin degradation through stabilization of Axin in colon cancer cells, and indicate that tankyrases are core components of the canonical Wnt pathway. Thus, it is thought that the application of tankyrase inhibitors may be broadly useful as antagonists of Wnt signalling [27], [28]. Indeed, several groups have undertaken structural studies to better understand how chemical inhibitors bind to tankyrases with the goal of designing more specific and effective compounds for therapeutic intervention [24], [31], [32], [33], [34].

**Table 1:** Sequences of primers used for Real-Time PCR.

Gene	Forward	Reverse
AXIN1	GGAGAGCGTGCAGGTCAAT	CACAGCCCATGTCCACACA
AXIN2	AGAAATGCATCGCAGTGTGAAG	GGGTTCTCGGGAAATGAGGTA
CTNNB1	TACCTCCCAAGTCCTGTATGAG	TGAGCAGCATCAAACTGTGTAG
HPRT1	ATGGACAGGACTGAACGTCTTGCT	TTGAGCACACAGAGGGCTACAATG
NKD1	TGAGAAGAAGATGGAGAGAGTGAGCGA	GGTGACCTTGCCGTTGTTGTCAAA
TNKS1	AGTGCTGTGGATATGGCTCCAACT	AGTCTGCTTCTCTGGCTGCTTGTA
TNKS2	TCATCTACAAACCTGCCGCCTACT	TTTCGACATCTCCAGCCTTTGCAG

Despite the fact that the Wnt pathway is an attractive target for anti-cancer therapy, there are few small molecule Wnt inhibitors available. Previous studies have shown that tankyrase inhibitors abrogate Wnt signalling in colorectal cancer cells with mutant APC [21], [22]. The aim of this study was to determine whether inhibition of tankyrases in breast cancer cells, the majority of which have normal β-catenin and APC, also results in stabilization of Axin and attenuation of Wnt signalling and cell growth. Here, we show that XAV939 inhibits tankyrase activity, promotes Axin stabilization and is effective in attenuating Wnt signalling and blocking cell migration in breast cancer cell lines. While XAV939 can also inhibit cell growth, this only occurs under conditions of serum-deprivation. These results thus suggest that application of tankyrase inhibitors for cancer treatment may require a combination strategy.

**Figure 1:**
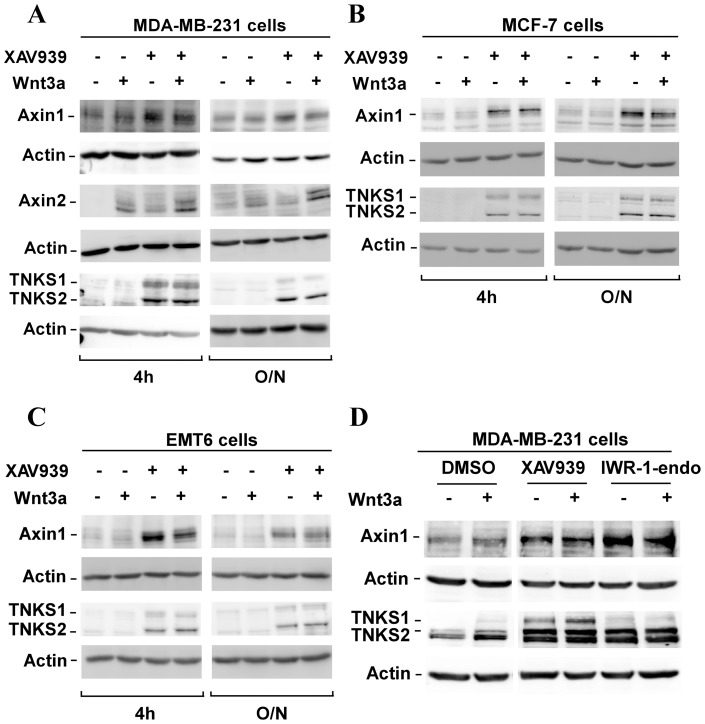
XAV939, a chemical inhibitor of tankyrases, stabilizes Axin in breast cancer cells. (**A–C**) Human MDA-MB-231 and MCF-7 cells and mouse EMT6 cells were treated overnight with 10 µM XAV939 in the presence or in the absence of Wnt3a conditioned medium either overnight or for 4 h and protein levels of Axin1, Axin2 and tankyrases (TNKS) were determined by immunoblotting of total cell lysates. (**D**) MDA-MB-231 cells were treated overnight with 10 µM XAV939 and IWR-1-endo and after 4 h incubation with Wnt3a conditioned medium, cells were lysed and subjected to immunoblot analysis using anti-Axin1, anti-Axin2 and anti-Tankyrase antibodies. All samples were also probed with anti-actin antibody to verify equal protein loading.

## Materials and Methods

### Compounds

XAV939 and compound 21H7 were purchased from Ryan Scientific, Inc (Mount Pleasant, SC, USA). IWR-1-endo was synthesized by the OICR Medicinal Chemistry Group (Toronto, ON, Canada).

**Figure 2:**
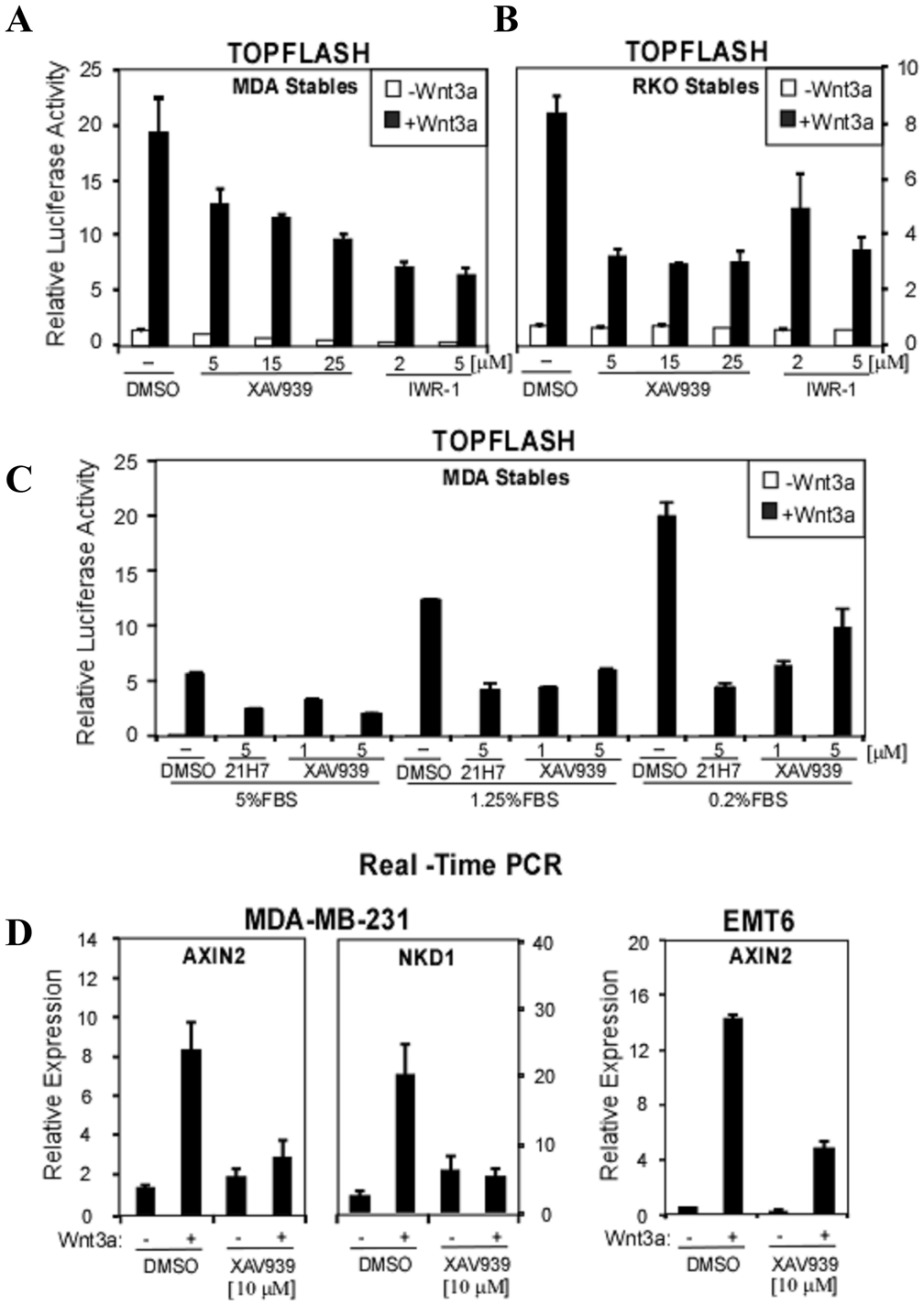
XAV939 blocks Wnt/β-catenin signalling in breast cancer cells. (**A–B**) Tankyrase inhibitors, XAV939 and IWR-1-endo, attenuate TOPFLASH activity in breast cancer MDA-MB-231 cells and in colon cancer RKO cells. MDA-MB-231 and RKO cells, stably expressing the Wnt-responsive TOPFLASH/pBAR and the control Renilla Luciferase reporter were treated with XAV939 and IWR-1-endo at the indicated concentrations in serum depleted medium (0.2% FBS) followed by 4 h incubation with control or Wnt3a conditioned medium. Cells were lysed and luciferase activities were measured. Data represents the mean of three replicates +/− standard deviation. (**C**) Inhibition of TOPFLASH activity by XAV939 in MDA-MB-231 stable cells maintained in medium supplemented with different FBS concentrations. Cells were treated overnight with 5 µM 21H7 and 1 or 5 µM XAV939 in the presence of control or Wnt3a conditioned medium with 5%, 1.25% or 0.2% FBS. Promoter activity was measured by luciferase assay. Data is shown as the mean of three replicates +/− standard deviation. (**D**) XAV939 decreases expression of Wnt target genes. MDA-MB-231 and EMT6 cells were treated with 10 μM XAV939 and Wnt3a-conditioned media overnight and the expression of Wnt target genes AXIN2 and NKD1 was measured by Real-Time PCR. Relative gene expression is plotted as the average of three PCR replicates +/− the range.

### Cell culture

All cell lines were obtained from American Type Culture Collection (ATCC) and were maintained according to ATCC recommendations. Human colon cancer cells, SW480 were cultured in alpha-MEM, DLD-1 in RPMI-1640 and RKO in EMEM. Human embryonic kidney cells, HEK293T, and mouse mammary tumor cells, EMT6, were grown in DMEM. Human breast cancer cell line, MCF-7, was maintained in DMEM supplemented with 1% non-essential amino acids (NEAA, Gibco). Human breast cancer cells DU4475 were cultured in RPMI-1640. All media were supplemented with 10% fetal bovine serum (FBS) (Hyclone, Thermo Scientific, Canada). Human breast cancer cells, MDA-MB-231, were maintained in RPMI-1640 supplemented with 5% FBS. MDA pBAR/Ren (MDA-MB-231 stable) and RKO pBAR/Ren (RKO stable) stably expressing the BAR reporter were generated by lentiviral transduction as previously described [35] where pBAR is a lentiviral plasmid that contains 12 TCF/LEF binding sites separated by distinct 5 base pair linkers upstream of a minimal promoter and the firefly luciferase open reading frame. BAR luciferase cell lines were also infected with a lentivirus carrying Renilla luciferase driven by a constitutive EF1-α promoter. MDA-MB-231 stable cells were maintained in RPMI-1640 supplemented with 5% FBS and 0.8 μg/ml puromycin (Sigma-Aldrich, Burlington, Canada) and RKO stable cells were maintained in DMEM supplemented with 10% FBS and 2 μg/ml puromycin. All cultures were routinely tested and found to be free of mycoplasma contamination. Wnt3a conditioned medium was prepared by collecting the supernatant from mouse L cells stably expressing murine Wnt3a gene as described previously [36], [37].

**Figure 3:**
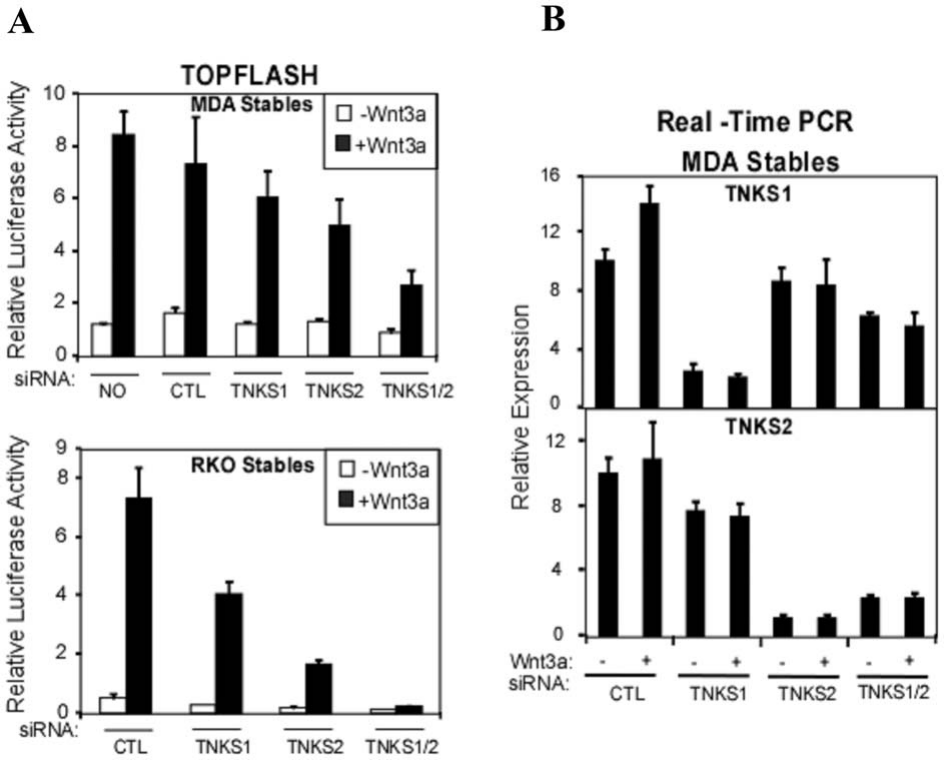
Knockdown of tankyrases inhibits Wnt/β-catenin signalling. (**A**) MDA-MB-231 and RKO stable cells were transfected with 20 nM siControl (siCTL) or siTankyrases 1 (TNKS1), siTankyrases 2 (TNKS2) or both. After 48 h, cells were cultured in the presence of control or Wnt3a conditioned medium for 4 h and were lysed. TOPFLASH activity was measured by luciferase assay and Firefly luciferase activity was normalized to Renilla luciferase activity, used as internal control. Values are shown as the mean of three replicates +/− standard deviations. (**B**) RNA was isolated from MDA-MB-231 cells, transfected with siCTL, siTankyrase 1, siTankyrase 2 or siTankyrase 1/2, and was subjected to Real Time PCR analysis to confirm knockdown efficiency. Relative gene expression is plotted as the average of three PCR replicates +/− the range.

### Transfections and Reporter assays

Cells were transfected using Lipofectamine 2000 (Invitrogen, Canada). For small interfering RNA (siRNA) silencing, cells were transfected with siRNA (Thermo Fisher Scientific, Dharmacon, Lafayette, CO, USA) against tankyrase 1 and tankyrase 2 for 48 h according to the manufacturer's protocol.

**Figure 4:**
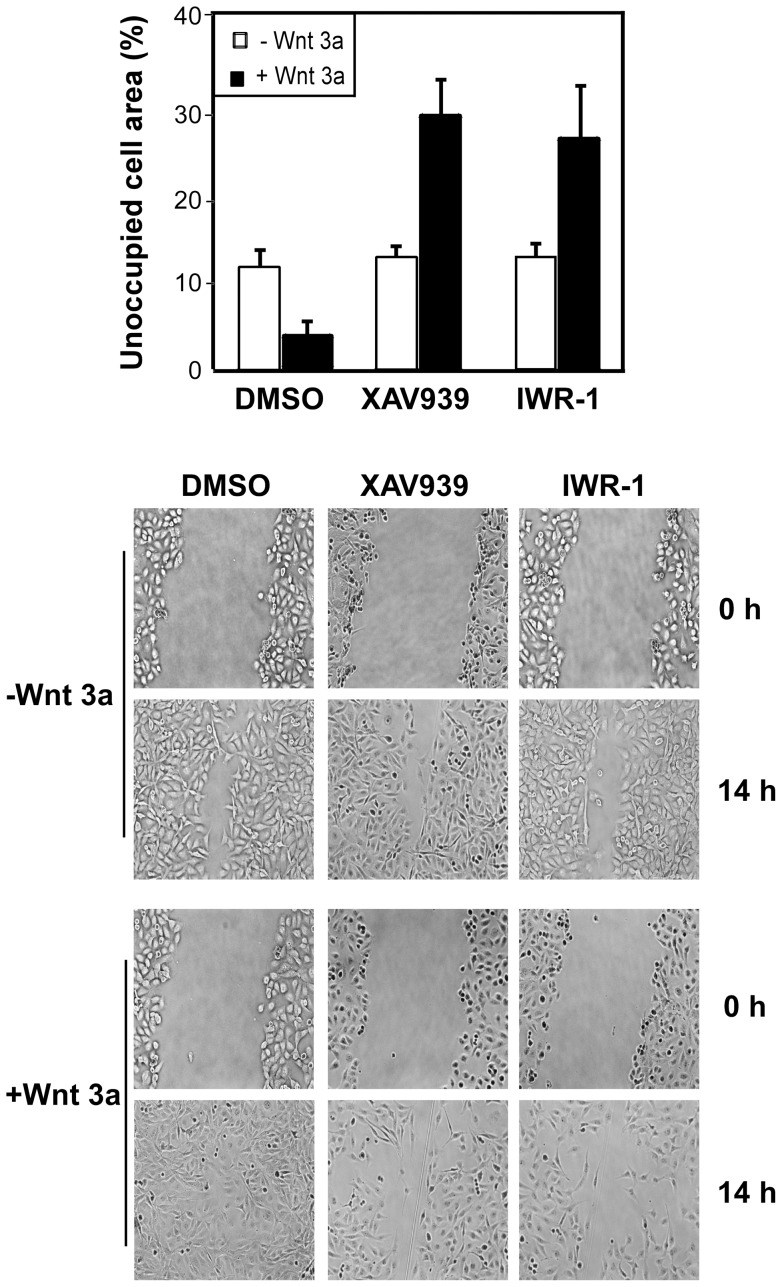
XAV939 inhibits migration of MDA-MB-231 cells. (**A**) A wound was introduced to confluent MDA-MB-231 monolayers and cells were then treated with 5 µM XAV939, 5 µM IWR-1 or DMSO in the presence of control or Wnt3a conditioned medium, supplemented with 2.5% FBS. A plot of the cell-free area within the wound, determined using Image J, is presented as percentage of the concurrent control at 0 h. (**B**) Representative microphotographs at 0 h and 14 h after wounding are shown.

For overnight incubation with compounds, HEK293T, MDA-MB-231 stable and RKO stable cells were treated with XAV939, 21H7 or DMSO in a serum-depleted medium for 1 h prior to addition of control or Wnt3a conditioned medium. In some experiments, cells were treated with compounds for 19 h prior to incubation for 4 h with control and Wnt3a conditioned medium as indicated. For colon cancer SW480 and DLD-1 cells compounds were added 5 h post-transfection and were incubated for 24 h in full serum containing medium.

**Figure 5:**
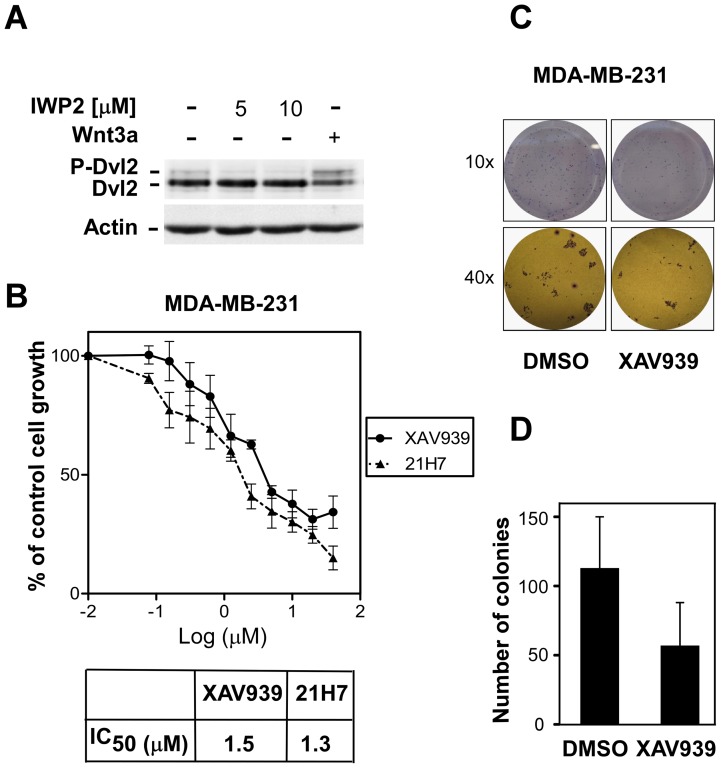
XAV939 suppresses growth and colony formation in MDA-MB-231 cells in low serum conditions. (**A**) MDA-MB-231 cells were treated overnight with the indicated concentrations of IWP-2 or with Wnt3a conditioned medium for 24h. Cell lysates were subjected to immunoblotting with Dvl2 antibody. Equal protein loading was verified by immunoblotting with anti-actin antibody. (**B**) MDA-MB-231 cells were treated for 72 h with the indicated concentrations of XAV939 or 21H7 in medium with low serum (1.25% FBS) and cell growth was determined by SRB assay. IC_50_ values for growth inhibition by XAV939 and 21H7 were calculated using GraphPad Prism 5.0 software and are shown (lower panel). (**C and D**) XAV939 impairs cell colony formation in MDA-MB-231 cells grown in low serum (1.25% FBS) conditions. Representative photographs are shown (**C**) Colony numbers were counted and a plot showing the average of 2 independent experiments +/− standard deviations is shown (**D**).

For TOPFLASH reporter assays, MDA-MB-231 stable and RKO stable cells were lysed and luciferase activity measured using the Dual Luciferase Reporter Assay System (Promega, Madison, MI) on a microplate luminometer (EG&G Berthold LB96V). Firefly luciferase activity was normalized to Renilla Luciferase activity used as an internal control.

**Figure 6:**
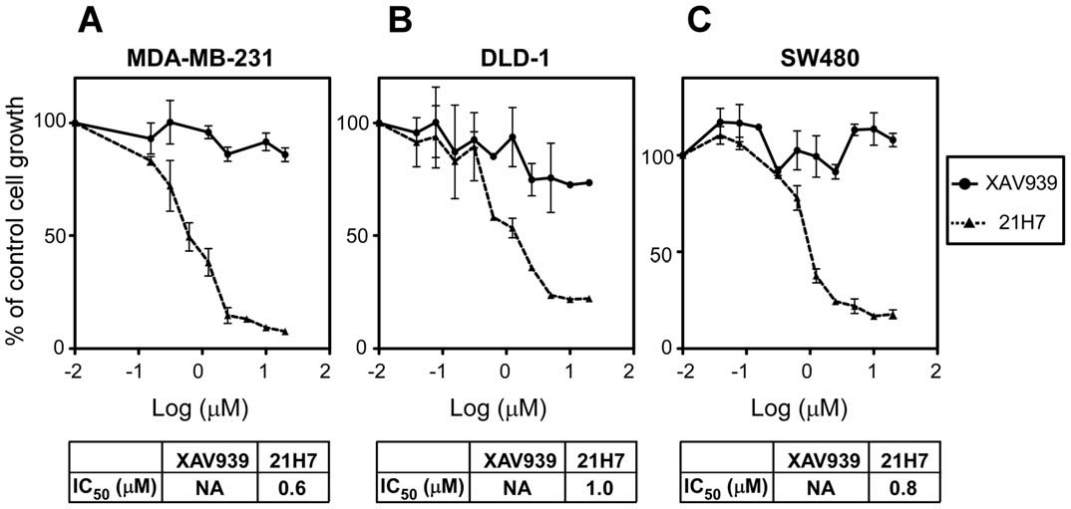
XAV939 has no effect on growth of cancer cells in optimal growth conditions. Growth inhibition was determined in human breast cancer cells MDA-MB-231 (**A**) and in human colon cancer cells DLD-1 (**B**) and SW480 (**C**) following treatment with XAV939 or 21H7 for 72 h. Cells were maintained in media with optimal serum concentration (5% FBS for MDA-MB-231 cells and 10% FBS for DLD-1 and SW480 cells). Under these conditions 21H7 potently decreases growth of breast and colon cancer cells whereas XAV939 has no effect. Graphs represent mean values +/− standard deviations from three independent experiments done in triplicate. IC_50_ values for growth inhibition (where applicable) are shown.

### Immunoblotting

Cells were lysed in non-denaturing buffer (50 mM Tris-Cl pH 7.4,150 mM NaCl, 1mM EDTA, 0.5% Triton X-100 supplemented with complete protease inhibitors, 10 µg/ml pepstatin A, 0.1 mg/ml trypsin inhibitor, 1 mM PMSF, 1 mM sodium vanadate, 50 mM NaF and 10 mM sodium pyrophosphate). Total protein content was measured by the Bradford assay (BioRad, USA) and equal amounts of protein were loaded on SDS-PAGE gels. For immunoblotting, the following antibodies were used: anti-Axin1 (R&D systems), anti-Axin2 (Cell Signaling Technology), anti-tankyrases (Abcam), anti-β-catenin (BD Biosciences), anti-Disheveled-2 (Dvl2) (Santa Cruz Biotechnology, Inc, Santa Cruz, USA) and anti-actin (Sigma-Aldrich, Burlington, Canada). The membranes were then incubated with HRP-conjugated secondary antibodies and bands were visualized by chemiluminescence.

### Quantitative Real-time PCR

Total RNA was isolated using the RNeasy Mini Kits (Qiagen, Mississauga, ON, Canada) according to the manufacturer's instructions. cDNA was generated from the purified mRNA using oligo-dT primer and reverse transcriptase (Fermentas, Burlington, ON, Canada). Real-Time PCR was performed using the SYBR Green PCR master mix (Applied Biosystems, USA) on the ABI Prism 7900 HT system (Applied Biosystems. USA) using validated primers (Table 1) as previously described [38]. Gene expression was normalized to HPRT and relative quantitation was calculated by the ΔΔCt method.

### Cell proliferation and colony growth assays

Growth inhibition of test compounds was determined using the Sulforhodamine B (SRB) assay. Cells were plated overnight in 96-well dishes (2500 cells/well) prior to compound or DMSO addition and 72 h later were fixed with 10% (w/v) trichloroacetic acid and stained as published [39]. The amount of SRB present in each well was determined by optical density reading at 490 nm and the results were expressed as a percentage of control cell growth. Each compound was assayed in triplicate. The IC_50_ values were determined using Prism 5.0 (GraphPad software, La Jolla, CA, USA). To assay the effect of XAV939 on colony formation, cells were plated into 6 well dishes at a density of 500 cells/well and 24 h later were treated with 10 μM XAV939 or with DMSO. Cells were incubated 4 or 5 days and after washing once with PBS, were stained with crystal violet (0.2% crystal violet solution in buffered formalin) and were photographed.

### Wound healing and cell migration assay

For wound healing assays, cells were seeded in a 6-well plate and were grown to form a confluent monolayer. The wound was introduced by scraping with a sterile 10 μl pipette tip and cells were then treated with XAV939, IWR-1 or DMSO and were incubated in control or Wnt3a conditioned media supplemented with 2.5% FBS. The progress of wound closure was monitored by microphotographs at 10x magnification taken with Zeiss inverted microscope equipped with CCD camera (Hamamatsu Photonic Systems). The percentage of the open wound area (area not occupied by cells) was calculated using Image J (NIH, Bethesda, USA).

## Results

### XAV939 increases Axin levels in breast cancer cells

Inhibition or abrogation of expression of tankyrases in colorectal cancer cell lines was previously reported to result in stabilization of Axin protein [21]. We confirmed this observation in SW480 and DLD1 cells as well as in HEK293T cells (Fig. S1A–C) and next sought to determine whether a similar effect was observed in breast cancer cells. For this, we first selected MDA-MB-231 cells, which are a basal-like triple-negative breast cancer cell line [40] that are responsive to the addition of exogenous Wnt ligand but also display low-level autocrine Wnt signalling that promotes proliferation and migration [38], [41], [42]. The requirement for canonical Wnt signalling for *in vitro* and *in vivo* metastatic properties has also been reported [43], [44], [45], [46]. MDA-MB-231 cells were treated overnight with the tankyrase inhibitor, XAV939, in the presence or absence of Wnt3a either overnight (right panels) or for the last 4 h (left panels) (Fig. 1A). Analysis of protein levels in aliquots of total cell lysates by immunoblotting revealed that Axin1 levels increased in MDA-MB-231 cells treated with XAV939 irrespective of the presence or absence of Wnt3a (Fig. 1A). Consistent with previous reports [21], XAV939 also increased the levels of both tankyrase 1 and 2, presumably by inhibiting auto-degradation [21]. Increases in the protein levels of the Wnt-inducible target gene, Axin2 were also observed in cells incubated with either Wnt3a or XAV939 and this was further enhanced in the presence of both treatments (Fig. 1A). We next examined the effect of tankyrase inhibitors in two other breast cancer cell lines, MCF-7 cells which are human epithelial-like cells of the Luminal A subtype that lack autocrine Wnt but are responsive to exogenous ligand [41], [44], [47] and EMT6 cells, a sarcoma-like mouse mammary breast cancer cell line, that has been utilized widely as a model system to study the effects of various treatments on local tumor growth and pulmonary metastasis [48], [49], [50]. A similar stabilization of Axin1 and tankyrase 1 and 2 by XAV939 was observed both in human MCF-7 and murine EMT6 cells (Fig. 1B and C). In these cells, Axin2 protein levels were not detectable and thus are not shown. Investigation of the effects of IWR-1-endo, a distinct tankyrases inhibitor, [21], [22] in MDA-MB-231 cells also resulted in stabilization of Axin1, tankyrase 1 and 2 (Fig. 1D). Mutations in APC that result in constitutive Wnt signalling in breast cancer cells are rare, nevertheless we tested the effect of XAV939 and IWR-1 in DU4475 cells, which harbor an APC mutation [47]. As for colon cancer cells with APC mutations (Fig. S1A and B), XAV939 induces stabilization of Axin1 as well as TNKS1 and 2 (Fig. S1D). Altogether, these results show that chemical inhibitors of tankyrases promote Axin stabilization in diverse human and mouse mammary tumor cell lines.

### XAV939 inhibition of Wnt signaling in breast cancer cells

We next examined whether inhibition of tankyrases alters Wnt-induced transcriptional activity in breast cancer cells. For this, we generated MDA-MB-231 cells stably expressing a Wnt-responsive TOPFLASH Firefly luciferase reporter along with a control Renilla luciferase reporter for normalization as previously described [51]. Treatment of cells with Wnt3a for 4 h (Fig. 2A) or overnight (Fig. 2C) following an overnight incubation in low serum conditions (0.2%) potently induced the TOPFLASH reporter and addition of XAV939 or IWR-1-endo inhibited this effect (Fig. 2A). Similar results were observed in a positive control line, the Wnt-responsive RKO colorectal cancer cells that also stably express the TOPFLASH reporter (Fig. 2B). Although slightly less potent, XAV939 also inhibited TOPFLASH reporter activity in MDA-MB-231 cells maintained in media containing 1.25% or 5% serum (Fig. 2C).

We next examined the effect of inhibiting tankyrases on the expression of endogenous Wnt-regulated target genes, Axin2 and Nkd1 by Real-Time PCR. Consistent with the TOPFLASH reporter assay, XAV939 inhibited Wnt-induced expression of Axin2 and Nkd1 in MDA–MB-231 cells (Fig. 2D). A similar reduction in Axin2 expression was observed in the mouse mammary cell line, EMT6 (Fig. 2D).

We next sought to confirm that it is XAV939 and IWR-1-endo-mediated inhibition of tankyrases that results in the inhibition of Wnt signalling using an alternative approach. For this, we abrogated expression of endogenous tankyrases in MDA-MB-231 breast cancer cells using siRNAs to ascertain if this had the same effect as the compounds (Fig. 3A). Tankyrase knockdown efficiency was confirmed using Real-Time PCR (Fig. 3B). While loss of either tankyrase 1 or tankyrase 2 alone gave only a slight decrease in Wnt3a-induced TOPFLASH signalling, elimination of the expression of both tankyrases resulted in a marked inhibitory effect on Wnt signalling (Fig. 3B). In control RKO colorectal cancer cells a similar, though more potent inhibition of the TOPFLASH reporter was observed upon abrogation of tankyrase 1 or tankyrase 2 expression.

Altogether these results show that inhibiting tankyrase activity either using chemical inhibitors or by abrogation of the expression of tankyrases using siRNA, blocks Wnt transcriptional responses in breast cancer cells.

### XAV939 inhibits Wnt3A-induced cell migration in MDA-MB-231 breast cancer cells

Wnt3a can promote cell migration in MDA-MB-231 cells [45], thus we next examined whether inhibition of tankyrases alters this effect. For this, we examined migration of MDA-MB-231 cells incubated with or without Wnt3a in the presence or absence of XAV939 and IWR-1-endo, using the wound-healing assay. Quantification of the cell-free region in the wounded area at 14 h showed that XAV939 and IWR-1 have no effect in reducing basal cell motility but markedly attenuated Wnt3a induced migration (Fig. 4). A decrease in cell migration by XAV939 was detected within 8–10 hours of wounding and under conditions of higher (5%) serum concentrations (data not shown) suggesting the effect of XAV939 inhibition is independent of effects on cell growth (see Fig. 4, below).

### XAV939 inhibits the growth of MDA-MB-231 breast cancer cells

Although MDA-MB-231 cells are responsive to the addition of exogenous Wnt ligand, these cells display low level of constitutive Wnt signalling which is thought to drive cell proliferation [41], [42]. We confirmed that our cell line displays autocrine Wnt signalling by monitoring the levels of phosphorylated Dvl2 (P-Dvl2), an indicator of active Wnt signalling. Phosphorylated Dvl2, which is visualized by a decreased electrophoretic mobility, is clearly detected in untreated MDA-MB-231 cells, is enhanced by exongenous Wnt ligand addition and is eliminated by the addition of IWP-2 [22], a specific inhibitor of endogenous Wnt ligand secretion (Fig. 5A). Thus, we next investigated the effect of XAV939 on cell growth in MDA-MB-231 cells using the Sulforhodamine B (SRB) assay. One day after plating, there was no significant difference in growth between control and XAV939 treated cells (data not shown) but after three days, XAV939-mediated inhibition of cell growth was observed. Specifically, we found that XAV939 blocks growth with an IC_50_ 1.5 μM for XAV939 (Fig. 5B). Similarly, in colony formation assays, XAV939 treatment at either 10 μM (Fig. 5C) or 3.3 μM (not shown) decreased the number of colonies formed (Fig. 5C and D). Altogether our results demonstrate that blocking tankyrase activity can inhibit Wnt transcriptional responses and diminish cell migration, growth and colony formation of breast cancer cells.

In the above described cell and colony growth experiments, cells were maintained in low serum conditions of 1.25%, so we next examined the effect of XAV939 on the growth of MDA-MB-231 cells in optimal serum (5%) conditions. Although Wnt signalling was still inhibited at this serum concentration (Fig. 2A and B), as was cell migration (data not shown), surprisingly, XAV939 had no effect on cell growth as measured by the SRB assay (Fig. 6A). For comparison, we examined growth in the previously tested DLD-1 and SW480 colorectal cell lines, both of which have constitutive Wnt signalling. Unexpectedly, our analysis revealed that in these serum conditions, XAV939 did not alter growth in either of these lines (Fig. 6B and C). In contrast, the recently described iron chelator, 21H7, which inhibits constitutive Wnt signalling and growth in colorectal cancer cells [39] independent of tankyrase or Axin stabilization (Fig. S1), blocked MDA-MB-231 (Fig. 6A), DLD-1 and SW480 (Fig. 6B and C) cell growth with IC_50_s in the range of 0.6–1.0 μM, as expected [39]. The ability of 21H7 to block Wnt transcriptional responses was also confirmed (Fig. 2C). Consistent with our observations, the previously reported effect of XAV939 on colony formation in DLD-1 cells was determined in low serum conditions [21]. Moreover, the ability of the tankyrase inhibitor, IWR-1-endo to attenuate DLD-1 cell viability in 1% but not 5% serum was also recently described [52].

Altogether, these results suggest that inhibition of tankyrases by XAV939 stabilizes Axin protein and can block Wnt transcriptional responses at all serum concentrations but that the presence of serum can overcome the inhibitory effect of XAV939 on cell growth. Thus, chemical inhibition of tankyrase activity is ineffective in blocking these alternative pathways that promote cell growth *in vitro*.

## Discussion

Wnt ligands bind to receptors on the cell surface and thereby induce a signalling cascade that leads to β-catenin-dependent activation of gene transcription. Numerous cancers display constitutive activation of the Wnt pathway. This has been most firmly established in the gastrointestinal tract, where mutations in the Wnt pathway component, APC and to a lesser degree in β-catenin, activate signalling and are associated with the majority of colon cancers [1], [2], [3]. In contrast, in breast cancer cells, which often display elevated Wnt signalling, mutations in APC or β-catenin are rare [10], [11].

Axin is a key component of the β-catenin destruction complex and when overexpressed inhibits Wnt signalling. Tankyrases have been shown to maintain low levels of Axin protein, thus in this study we examined the effect of inhibiting tankyrase activity in breast cancer cells. Consistent with reported results on APC-mutant colorectal cancer cells [21], [22], tankyrase inhibitors stabilized Axin1 expression in DU4475 breast cancer cells, that harbor a mutant APC [47]. We also demonstrated that tankyrase inhibitors induce Axin1/2 stabilization in human MDA-MB-231 basal-like cells, human MCF-7 epithelial-like cells and mouse EMT6 sarcoma-like cells, all of which are breast cell lines of diverse origins but with normal APC. We also confirmed that a different tankyrase inhibitor, IWR-1-endo [21], [22] similarly promoted Axin1/2 stabilization in MDA-MB-231 cells. Concomitant with Axin stabilization, XAV939 inhibited Wnt-induced transcriptional responses as measured by the TOPFLASH reporter and by examining endogenous Wnt target gene expression. Abrogation of the expression of tankyrases using siRNA also attenuated Wnt signalling, suggesting that the compounds do indeed work through tankyrases. Altogether our study demonstrates that Axin stabilization via inhibition of tankyrases can inhibit Wnt signalling in certain breast cancer cells harboring normal APC. Moreover, our work serves to further highlight the importance of Axin as a key regulatory node in the Wnt signalling cascade.

An unexpected observation was that although tankyrases blocked cell growth in the breast cancer cell lines, the compound was only effective in low serum conditions. The ability of XAV939 to block cell growth and colony formation is prevented in the presence of optimal serum concentration even though Wnt-induced transcriptional responses were attenuated. In contrast, Wnt3a induced cell migration was attenuated by XAV939 even in the presence of high serum. Presumably, growth factors in full serum can compensate for the loss of β-catenin signalling to maintain cell growth but not cell migration. Besides inhibiting Wnt signalling, tankyrases have been reported to target multiple proteins to affect diverse cellular processes including telomere maintenance by interacting with TRF1, spindle formation and stabilization by binding to NuMA, glucose metabolism by regulating GLUT4 transport through IRAP [53], [54], [55] and an involvement in Cherubism disease due to dysregulation of 3BP2 degradation [54]. It may be that these or other uncharacterized activities provide an explanation for why inhibition of tankyrases is only effective in inhibiting cell growth at low serum even though Wnt transcriptional responses are attenuated. Studies by others using the compound IWR-1-endo also confirm that IWR-1-endo does not inhibit growth when the assay is performed using optimal serum [52]. Irrespective of the molecular explanation, these results indicate that stabilization of Axin by inhibition of tankyrases, may not be an effective means to block tumor cell growth and thus may not be appropriate as a stand-alone therapeutic approach. Rather, it may be necessary to use tankyrase inhibitors in combination with other pathway specific compounds to effectively attenuate tumor growth. Further investigation of the molecular mechanisms that regulate Axin protein levels via tankyrases and deregulation in disease conditions is likely to provide additional avenues for treating Wnt-dependent cancers and other Wnt-related diseases.

## Supporting Information

Figure S1 **XAV939 stabilizes Axin and Tankyrases.**
(PDF)
