# Supplementary material for: Inhibition of Tankyrases Induces Axin Stabilization and Blocks Wnt Signalling in Breast Cancer Cells
Source: PLoS One. 2012 Nov 7;7(11):e48670. doi: 10.1371/journal.pone.0048670 (PMC3492487; doi:10.1371/journal.pone.0048670)
Supplement: Figure S1 — XAV939 stabilizes Axin and Tankyrases. (PDF) [file pone.0048670.s001.pdf]

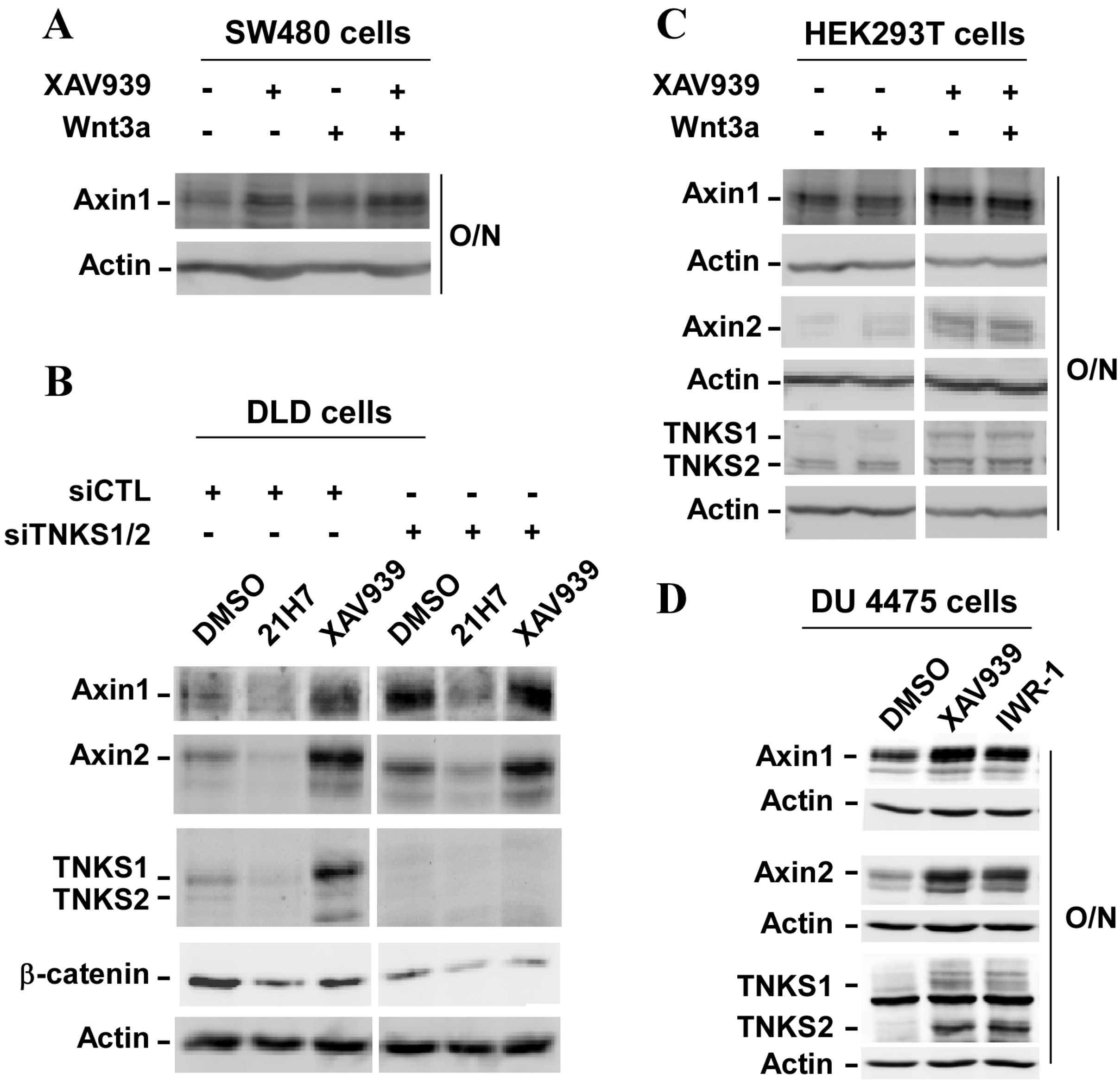

Figure S1. XAV939 stabilizes Axin and Tankyrases.
(A) SW480 cells were treated overnight with 5 μM XAV939 prior to the addition of control or Wnt3a conditioned medium for 4 h. Cell lysates were analyzed by immunoblotting with anti-Axin1 antibodies. **(B)** DLD-1 cells were transfected with 20 nM siTankyrases 1 and siTankyrases 2. Cells were then treated with 1 μM XAV939 or 21H7 for 16 h and were incubated in Wnt3a conditioned medium for 4 h. Cell lysates were analyzed by immunoblotting with anti-Axin1, anti-Axin2 and anti-β-catenin antibodies. Knockdown of tankyrases was confirmed by immunoblotting with anti-Tankyrase1/2 antibodies. **(C)** HEK293T cells were treated overrnight with 4 μM XAV939 in the presence of control or Wnt3a conditioned medium. Cell lysates were analyzed by immunoblotting with anti-Axin1, anti-Axin2, and anti-Tankyrase1/2 antibodies. **(D)** Human breast cancer DU 4475 cells,which harbor a mutation in APC, were incubated with 5 μM XAV939 or 5 μM IWR-1 overnight. Cell lysates were subjected to immunoblotting with anti-Axin1, anti-Axin2 and anti-Tankyrase 1/2 antibodies.**(A-D)** Equal protein loading was verified by immunoblotting with anti-actin antibody.
